# Redox-Enhanced Photoelectrochemical Activity in PHV/CdS Hybrid Film

**DOI:** 10.3390/nano13091515

**Published:** 2023-04-28

**Authors:** Mengyu Fu, Dongzi Xu, Xiaoxia Liu, Yuji Gao, Shenghong Yang, Huaifeng Li, Mingming Luan, Pingping Su, Nianxing Wang

**Affiliations:** 1 School of Chemistry and Pharmacy, Qilu University of Technology, Jinan 250353, China; 2 State Key Laboratory for Chemistry and Molecular Engineering of Medicinal Resources, School of Chemistry and Pharmaceutical Sciences, Guangxi Normal University, Guilin 541004, China; 3 Delsitech Ltd., Itäinen Pitkäkatu 4 C (PharmaCity), 20520 Turku, Finland

**Keywords:** CdS nanoparticles, viologen, photoelectrochemical redox materials, hybrid films

## Abstract

Semiconductive photocatalytic materials have received increasing attention recently due to their ability to transform solar energy into chemical fuels and photodegrade a wide range of pollutants. Among them, cadmium sulfide (CdS) nanoparticles have been extensively studied as semiconductive photocatalysts in previous studies on hydrogen generation and environmental purification due to their suitable bandgap and sensitive light response. However, the practical applications of CdS are limited by its low charge separation, which is caused by its weak ability to separate photo-generated electron-hole pairs. In order to enhance the photoelectrochemical activity of CdS, a polymer based on viologen (PHV) was utilized to create a series of PHV/CdS hybrid films so that the viologen unit could work as the electron acceptor to increase the charge separation. In this work, various electrochemical, spectroscopic, and microscopic methods were utilized to analyze the hybrid films, and the results indicated that introducing PHV can significantly improve the performance of CdS. The photoelectrochemical activities of the hybrid films were also evaluated at various ratios, and it was discovered that a PHV-to-CdS ratio of 2:1 was the ideal ratio for the hybrid films. In comparison with CdS nanoparticles, the PHV/CdS hybrid film has a relatively lower band gap, and it can inhibit the recombination of electrons and holes, enhancing its photoelectrochemical activities. All of these merits make the PHV/CdS hybrid film as a strong candidate for photocatalysis applications in the future.

## 1. Introduction

In recent years, research on photocatalysis using semiconductive materials has gained significant attention. Semiconductive photocatalytic nanoparticles have been widely used in a variety of applications, including energy storage, solar fuel generation, and pollutant degradation [1,2,3]. These photocatalysts offer numerous advantages, such as good physical/chemical stability, low cost, and environmental friendliness. Metallic or metalloid semiconductors, such as AgI, TiO_2_, ZnO, and SnO_2_, and sulfide semiconductors, such as ZnS and CdS, have been synthesized as photocatalytic materials [4,5,6,7]. Due to their strong light absorption ability, abundant active sites, and suitable band gaps, CdS nanoparticles have become one of the most promising photocatalysts and have been widely studied and utilized in photocatalytic applications [8,9,10,11].

The exceptional photocatalytic activity of CdS is based on its ideal band gap of 2.42 eV, which allows it to absorb visible light more efficiently than other photocatalytic materials. Furthermore, CdS has a negative conduction band, which is suited to the thermodynamic requirements of many photocatalytic reactions. On the other hand, its fast recombination rate strongly limits the efficiency of CdS, and also limits its applications [12,13,14]. Therefore, improving the photocatalytic efficiency of CdS is necessary to broaden its applications. Additionally, CdS not only has unique advantages as a green chemistry technology but it also has energy saving and environmentally friendly characteristics. In order to overcome its drawbacks and improve the performance of CdS, different inorganic and organic materials have been utilized to work as electron/hole acceptors to improve their photocatalytic performance [15,16,17,18]. With the assistance of different acceptor materials, both the photocatalytic efficiency and the energy efficiency of CdS are improved, and the utilization of green energy, such as solar energy, can be realized. In this work, a viologen-based polymer was introduced to form redox active hybrid films together with CdS, and with the assistance of viologen, the photoelectrochemical performance of CdS can be enhanced [3,19,20,21,22,23].

**Scheme 1:**
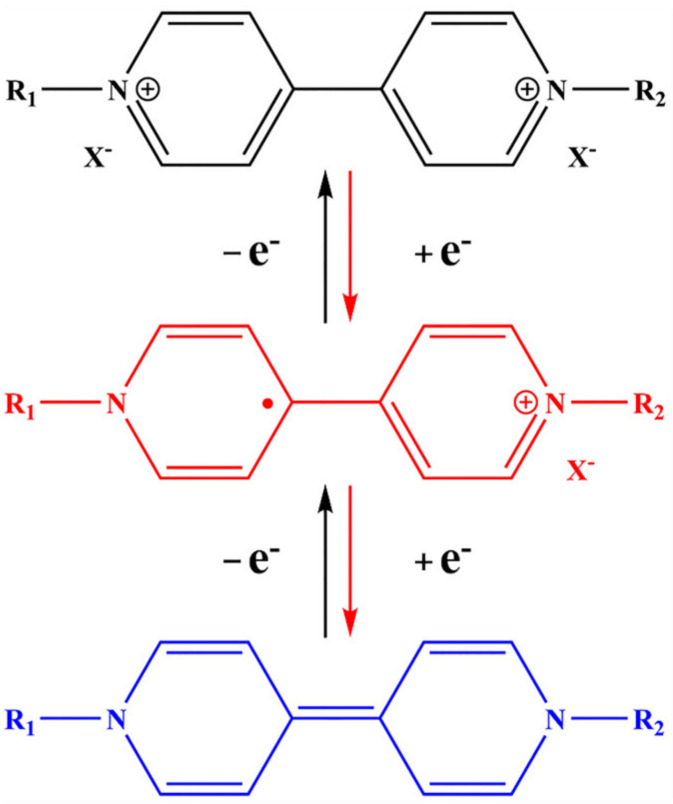
Three states of viologens during redox processes. From top to bottom: dication, radical cation, and neutral.

Viologens are organic compounds that contain 4′4-bipyridine structures, and their unique structures endow them with distinct redox properties [24,25]. Viologen can exhibit three different states: dication, radical cation, and neutral, as shown below in Scheme 1. Usually, viologen is in the dication state, which is the most stable of the three different states. When viologen accepts one electron, it is reduced into the radical cation state, and, correspondingly, viologen can switch back to the dication state when it loses one electron. As is shown in the first redox process in Scheme 1, this redox process is reversible and stable, so it enables viologens to be utilized as mediator materials in a variety of applications, including sensors, fuel cells, and redox flow batteries [26,27,28,29,30,31]. Additionally, the free electron transfer between the two pyridine rings enables viologens to exhibit intense colors in the radical cation state. The color of a viologen in its radical cation state can be modified by changing the substituted groups, and the reductive potentials will therefore be adjusted correspondingly. As a result, viologens have been widely applied in electrochromic devices and smart windows [32,33,34]. From their radical cation state, viologen can be further reduced into their neutral state. However, in some viologen materials this process is reversible, meaning that neutral state viologens can be oxidized back to the radical cation state, but in others it is irreversible. The second redox process is quite different from the materials. It has been proved that the substituted groups on the viologens could strongly influence the reversibility of the second redox process.

In previous years, our group synthesized various viologen-based materials with different substituted groups, all of which demonstrated good redox properties [35,36,37]. Traditionally, methyl viologen (MV) has been utilized to analyze the photochemical performance of different photocatalytic nanoparticles, such as TiO_2_, CdS, and so on [38,39,40]. Previous research on viologen materials in combination with CdS has primarily focused on the mechanism of electron transfer, with both materials typically dissolved in solution. For example, MV has been used in combination with CdS nanomaterials to study radical generation, and it has been shown that the presence of MV can effectively increase the electron transfer between MV and CdS [22,41,42,43]. On the other hand, photocatalytic research on MV together with CdS materials has also shown that MV can improve the photocatalytic efficiency of CdS [20,44]. However, in previous research, MV was typically used as the mediator, which is advantageous because of its stable redox property and its ability to lower the relative rate of charge recombination. The drawback of MV is that it is toxic, and since it is a small molecule it is prone to leakage into the environment. To address this issue, a viologen-based polymer, poly-hexenyl viologen (PHV), was used as the electron acceptor in combination with CdS to form redox-active hybrid films. Compared with MV, PHV has good stability, environmentally friendly properties, and a long chain, making it suitable not only as an electron acceptor but also for immobilizing CdS nanoparticles.

In this work, the CdS and PHV were synthesized and characterized using X-ray diffraction (XRD) and nuclear magnetic resonance (NMR), respectively. After this, they were utilized as the initial materials to form the PHV/CdS hybrid films. In the hybrid films, the CdS offers good photoactive properties, and the PHV works as the electron acceptor, enabling the hybrid films to obtain reversible redox properties. The hybrid films were obtained by mixing the solutions with different ratios of PHV and CdS, after which the generated films were characterized using various electrochemical and photoelectrochemical tests to find the one with the best performance. After comparing the results, we were able to determine that the best PHV-to-CdS ratio was 2:1. The PHV/CdS hybrid film with the best ratio was prepared and analyzed using electrochemical impedance spectroscopy (EIS), UV-vis, fluorescence spectroscopy, SEM, and TEM, and it was noticed that all of the hybrid materials had enhanced photochemical performance due to the PHV. Additionally, the band gap of the hybrid films was also improved compared with the CdS nanoparticles, and this broadens the application potential of the hybrid films. In general, PHV can work as a good electron acceptor to improve the photocurrent generation efficiency of CdS, i.e., to improve its photocatalysis abilities. This study provides a simple method for obtaining a PHV/CdS hybrid film with enhanced photoelectrochemical properties for use in photocatalytic applications in the future.

## 2. Materials and Methods

### 2.1. Chemicals

All the chemicals which were used in this work, such as KCl, CdCl_2_·2.5H_2_O, mercaptopropionic acid, NaOH, Na_2_S·9H_2_O, 4,4′-bypridine, dibromahexane, acetonitrile, Nafion, and so on, were obtained from Aladdin (Seattle, WA, USA). The KCl was used to prepare the electrolyte solution, and all the solutions used in the electrochemical experiments were prepared using deionized water and purged with N_2_ for 15 min before the experiments.

### 2.2. Preparation of CdS Nanomaterials

Add 35 mL ultrapure water to a 50 mL round-bottomed flask, dissolve 1 mmol CdCl_2_ 2.5H_2_O (228.4 mg) and 2 mmol mercaptopropionic acid (MPA), and stir until mixed well. To reduce the pH of the solution to about 7, add a 10 M NaOH solution dropwise with fast stirring. After refluxing for 30 min, add 5 mL Na_2_S·9H_2_O (1 mmol, 240.18 mg) to the mixture and stir for 3 h at room temperature to generate yellow and clear MPA–CdS nanomaterials.

### 2.3. Synthesis of PHV

The PHV was synthesized using the Menshutkin reaction in a 3-neck round bottom flask, in which 4,4′-biypridine (1.56 g, 10 mmol) and dibromahexane (2.44 g, 10 mmol) were dissolved and mixed with acetonitrile solution (ACN, 120 mL; Aldrich, Saint Louis, MI, USA, 99.9%). the solution was then refluxed at a temperature of 80 °C for 24 h. After this, the products were washed with ACN and dried at room temperature. The dialysis of the PHV was carried out using semipermeable membrane to obtain a high molecular weight fraction.

### 2.4. Preparation of PHV/CdS Hybrid Films

The PHV solution was mixed with CdS solutions with different concentration ratios (1:3, 1:2, 1:1, 2:1, 3:1, 4:1, 6:1, and 8:1). The PHV concentration was maintained at 5 mM in all solutions, and the CdS concentrations were changed in accordance with the ratios. The PHV/CdS mixed solutions were doped onto ITO glasses with a homemade template (5 mm Ø, 200 μL). After drying, the hybrid film was covered with Nafion solution (1%, 100 μL). In the PHV/CdS hybrid films, the PHV content was always kept at 1 millimole, and the amounts of CdS (20.7 millimoles, 13.8 millimoles, 6.9 millimoles, 3.4 millimoles, 2.3 millimoles, 1.7 millimoles, 1.1 millimoles, and 0.86 millimoles) were added so that the CdS concentrations would correspond to the concentration ratios (1:3, 1:2, 1:1, 2:1, 3:1, 4:1, 6:1, and 8:1).

### 2.5. XRD and NMR Measurement

The prepared CdS nanoparticles and PHV polymers were analyzed using XRD and NMR, respectively. The CdS was analyzed using a Bruker, Billerica, MA, USA, D8 ADVANCE XRD operating at 35 kV and 40 mA. The XRD spectra were recorded in a 2θ range of 5–80° with a scanning rate of 20°/min. The 1H NMR spectra were recorded using a Bruker Advance III 400 MHz spectrometer at room temperature, and the chemical shifts were referenced to the residual proton resonances.

### 2.6. Electrochemical Methods

All the electrochemical experiments were performed using an Ivium (Eindhoven, The Netherlands), electrochemical workstation (Iviumstat.h) and its accessories in different three-electrode cells. A glassy carbon electrode (3 mm Ø) and an ITO glass (8 Ω, 5 mm Ø) were used as working electrodes. The Ag/AgCl electrode (3.5 M KCl) was used as the reference electrode and the Pt wire was used as the counter electrode. Both the PHV and PHV/CdS hybrid films were analyzed using cyclic voltammetry (CV) from 0 to −1.2 V for 3 cycles. The scan rate was shifted from 5 mV/s to 200 mV/s, and 0.1 M KCl was used as the electrolyte. The photochemical performance of PHV/CdS hybrid films was also assessed using an LED light source (Modulight, Ivium) that emitted UV light (365 nm, 20 mW/cm^2^) and blue light (460 nm, 20 mW/cm^2^) in 0.1 M KCl solution. The EIS measurement was carried out using an Iviumstat.h, the frequency scan was from 10,000,000 Hz to 1 Hz, the frequencies each decade were 5, the frequencies were 36, and the amplitude was 0.01 V. A glassy carbon electrode was used as the working electrode, and 0.1 M KCl solution was used as the electrolyte.

### 2.7. Spectroscopic and Microscopic Methods

UV-vis and fluorescence spectroscopy were utilized to analyze the CdS nanoparticles and the PHV/CdS hybrid films. The UV-vis spectra were obtained using a Shimadzu UV-2600PC spectrophotometer (Shimadzu Corp., Kyoto, Japan), and the measurement range was from 200 nm to 600 nm. The Fluorescence spectra were measured using an F97Pro spectrophotometer (Lengguang Tech., Shanghai, China). The excitation wavelength of the fluorescence measurements was 380 nm, the scanning speed was 3000 nm/min, the scanning interval was 1 nm, the excitation bandwidth was 10 nm, and the emission bandwidth was 10 nm. In order to analyze the morphology of the synthesized CdS particles, transmission electron microscopy (TEM) was used, and carbon foils were used as the supports for the TEM samples. TEM images of the CdS nanoparticles were taken using a Tecnai G2 20 TWIN transmission electron microscope (Hillsboro, OR, USA). The PHV/CdS hybrid films were analyzed using a scanning electron microscope (SEM), and ITO glasses were used as the template. SEM was used to measure the morphology of the CdS and PHV/CdS hybrid films, and images were acquired using a Quanta 200FEG SEM spectrometer at 20 kV, 10 mA, SE mode, and spot size of 3.5 (Hillsboro, OR, USA).

## 3. Results and Discussion

**Figure 1:**
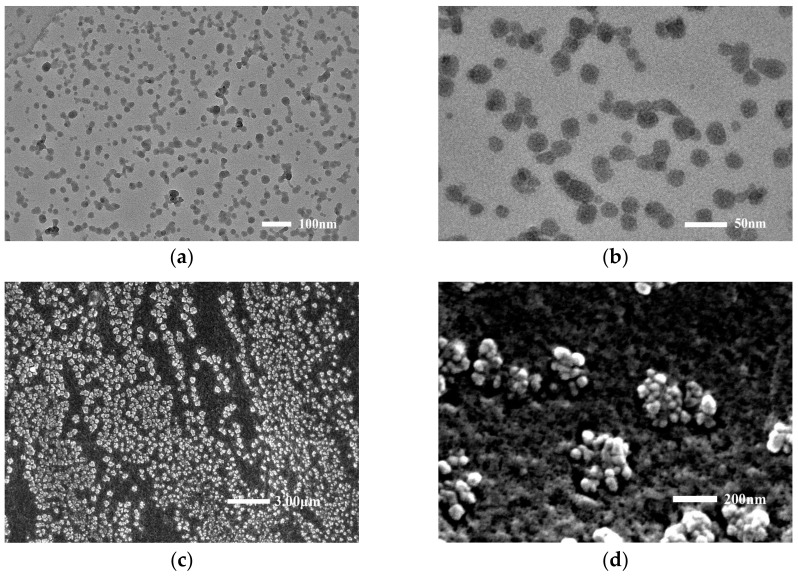
(**a**,**b**) TEM images of CdS. (**c**,**d**) SEM images of CdS.

The photoelectrochemical performance of CdS nanoparticles is greatly influenced by their morphology; therefore, it is essential that the CdS be manufactured at appropriate sizes. A transmission electron microscope (TEM) and a scanning electron microscope (SEM) were used to study the morphologies and sizes of the artificially produced CdS nanoparticles. The TEM images shown in Figure 1a,b demonstrate the presence of CdS nanoparticles on the carbon foil. It can be observed that the CdS nanoparticles had been properly synthesized, and that their sizes roughly ranged from 20 nm to 30 nm, making them quite uniform. In the SEM images shown in Figure 1c,d, the CdS nanoparticles appear evenly distributed throughout the materials, allowing for effective dissemination of the solutions. Additionally, X-ray powder diffraction (XRD) was also utilized to characterize the synthesized CdS nanoparticles, as can be observed in the Supplementary Information in Figure S1. The XRD pattern shows a broad peak at 26.5°, corresponding to the (100), (002), and (101) planes of the CdS nanoparticles. The medium diffraction peaks at 44.0° and 51.5° can be ascribed to the (110) and (112) planes of the CdS nanoparticles, respectively. There are several more weak diffraction peaks that can be found at 33.7°, 64.5°, and 70.6°, and these may have been caused by irregular CdS crystals produced during synthesis. All the results indicated that the CdS nanoparticles were successfully synthesized [45,46].

**Figure 2:**
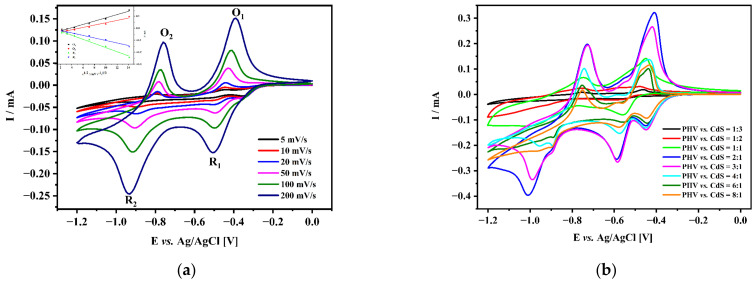
The cyclic voltammetry measurements of (**a**) PHV and (**b**) PHV/CdS hybrid films.

The synthesized PHV was characterized using nuclear magnetic resonance (NMR), and the result are shown in Figure S2. The doublet resonances at 9.11 ppm and 8.54 ppm can be ascribed to the H of the pyridinium rings in the long polymeric chain. The triblet resonance at 4.73 ppm and the multiplet resonance at 2.09 ppm can be ascribed to the H of the CH_2_ alkyl groups attached to the pyridine N in the long polymeric chain. After the synthesis of the PHV, in order to characterize its redox property and that of the PHV/CdS hybrid films, cyclic voltammetry (CV) was utilized. The redox performance of the PHV in 0.1 M KCl electrolyte was analyzed using CV from 0 V to −1.2 V with various scan rates, as can be seen in Figure 2a. It can be observed that the two redox couples are clearly shown in the CVs, and the peak values increased in accordance with the scan rates increments. The first reductive peak (R_1_) can be found at approximately −0.50 V, and the corresponding oxidative peak (O_1_) can be found at approximately −0.39 V and was caused by the changes in the state of the viologen from the dication state to the radical cation state, and back again. For the same reason, the second redox couple can be observed at approximately −0.93 V (R_2_) and −0.75 V (O_2_), and these were formed due to the viologen being switched from the radical cation form to the neutral form and back again. The figure inserted in the top-left of Figure 2a shows the peak currents at each reductive/oxidative peak under various scan rates vs. the square root of the scan rate, and it can be seen that the peak currents are proportional to the square roots of the scan rates. Based on the Randles–Sevcik equation, we can know that the redox properties of the PHV films are reversible.

After the characterizations of the CdS and PHV, the hybrid films were prepared with different ratios, as described above, and the hybrid films were also analyzed using the CV method. The hybrid films were measured in 0.1 M KCl electrolyte with the potential ranging from 0 V to −1.2 V, and the scan rate was carried with 50 mV/s. The redox performances of the various PHV/CdS hybrid films are shown in Figure 2b above. The concentration of the initial solutions was mixed with PHV and CdS at different ratios from 1:3 to 8:1, and it can be observed that all the hybrid films show the typical redox couples from viologen. When the CdS concentration was high, the redox responses of the hybrid films were fairly weak because there were too many CdS nanoparticles, so the aggregation was formed and the electron transfer between the PHV was prevented. However, when the PHV concentrations were reduced, the peak currents of the hybrid films increased, meaning that the redox behavior of the PHV became the dominant part of the hybrid films. In comparing all the PHV/CdS hybrid films, the PHV-to-CdS ratio of 2:1 had the best performance, indicating that this ratio is the best for the hybrid film. In this ratio, the CdS nanoparticles can be immobilized by the PHV, and the PHV is also able to produce the best redox performance. Additionally, an extra reductive peak was observed at approximately −0.4 V, and the reductive peaks from the PHV were shifted to approximately −0.6 V and −1.0 V. It is assumed that this was caused by the participation of the CdS nanoparticles in the hybrid films, which improved the electron transfer within the hybrid films.

**Figure 3:**
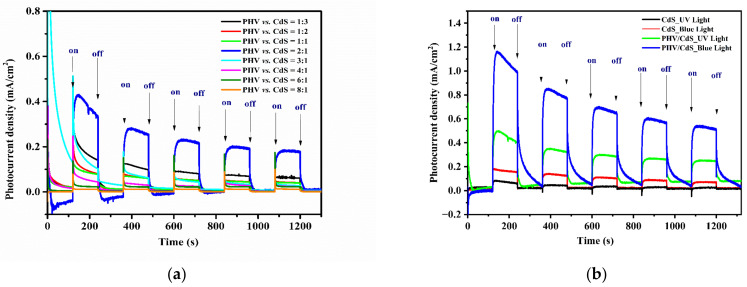
The photoelectrochemical measurements of (**a**) various PHV/CdS hybrid films and (**b**) CdS and PHV/CdS hybrid films.

In order to analyze the photocatalytic performances of the PHV/CdS hybrid films, an Ivium modulight with UV light was utilized to measure the photocurrents of the various hybrid films, and the results are shown in Figure 3a below. It can be observed that all the hybrid films with different ratios showed good photoelectrochemical performance under UV irradiation, and that by adjusting the PHV-to-CdS ratios the photocurrent value can be changed. When the CdS concentration was high, e.g., 1:3, the photocurrent was relatively high, but this might only have been caused by the CdS nanoparticles because the photocurrent values were reduced when the CdS concentration decreased. Of all the hybrid films, the one prepared with the PHV-to-CdS ratio of 2:1 achieved the highest photoelectrochemical measurement value. This was because the good redox property of the PHV enabled it to rapidly obtain the photoelectrons which were generated by the CdS nanoparticles, increasing the charge separation and thereby enhancing the photoelectrochemical performance of the hybrid film. This result is consistent with the result of the above CV test, proving that the 2:1 ratio is the best ratio for PHV/CdS hybrid films. We can also assume that the CdS nanoparticles were distributed well in the hybrid film. Subsequently, when the CdS concentration was decreased continuously in the other hybrid films, the photocurrent started to reduce again. Since the 2:1 PHV/CdS hybrid film had the best performance, which was consistent with previous CV measurements, we are able to confirm that the best ratio for a PHV/CdS hybrid film is 2:1, and in the following experiments, all the PHV/CdS hybrid films were prepared according to the ratio 2:1.

In addition to the photoelectrochemical measurements with UV light, visible light (blue light) was also utilized to test the photocatalytic properties of the CdS and PHV/CdS (2:1) hybrid films. In Figure 3b above, it can be observed that when the PHV was added to the CdS to form a hybrid film, the photocurrent values increased significantly, indicating that their photoelectrochemical performance had been effectively improved. This can be explained by the good redox properties of the PHV, which can accept the photogenerated electrons from the CdS quickly and effectively, improving the photocatalytic properties of the PHV/CdS correspondingly. Moreover, both the CdS and PHV/CdS hybrid films exhibited enhanced photocurrent values under blue light irradiation, indicating their potential for visible light photocatalytic applications.

**Figure 4:**
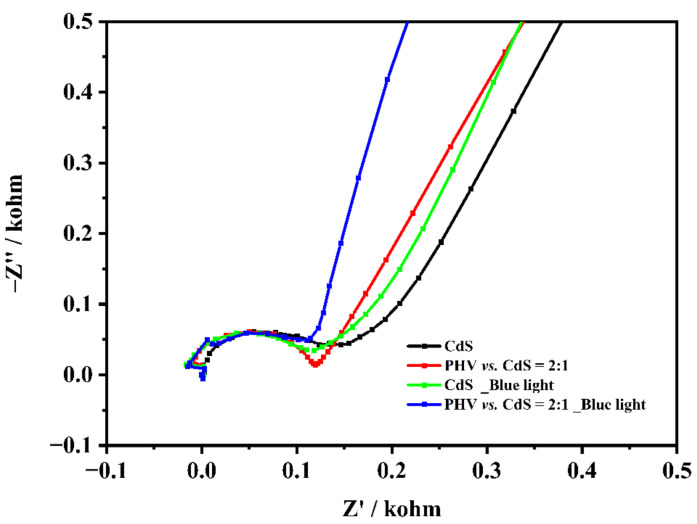
The EIS measurement of CdS nanoparticles and PHV/CdS hybrid film (2:1) with/without light irradiation.

In order to further analyze the photoelectrochemical performance of the CdS nanoparticles and the hybrid film (PHV vs. CdS = 2:1), electrochemical impedance spectroscopy (EIS) was utilized to characterize the charge transfer mechanism under blue light irradiation. In Figure 4 below, the EIS measurements of the CdS nanoparticles and the PHV/CdS hybrid film are shown with and without blue light irradiation. It can be observed that the Nyquist semicircle of the CdS was the largest, meaning that its conductance was the lowest. However, the EIS measurements of the prepared hybrid film resulted in a smaller semicircle for the PHV/CdS, meaning that the interfacial conductivity was increased by the PHV. On the other hand, when blue light irradiation was applied, both the CdS and PHV/CdS showed improved EIS performance, which can be explained by the photoelectrons that were generated, increasing their conductances. In contrast, the performance of the PHV/CdS was significantly improved because the PHV can work as a good electron acceptor, thus suppressing the recombination of the photo-generated electrons. Based on these results, it can be concluded that the PHV/CdS can acquire better charge transfer properties under light irradiation. Thus, the hybrid film has promising prospects in photoelectrochemical applications.

The reusability of the PHV/CdS hybrid films was also analyzed using cyclic voltammetry, as is shown in Figure S3. The redox properties of the PHV/CdS hybrid films with the 2:1 ratio were measured before and after a series of photoelectrochemical blue light tests. It can be observed that after the first photochemical test, the peak currents of the hybrid film slightly decreased because some of the materials that were not well immobilized by the Nafion had dropped into the solution. The same phenomenon was also observed after the second photochemical test. However, after the third and fourth tests, there was no significant change in the redox response between the CVs, indicating that the PHV/CdS hybrid films had good reusability and could be reused many times in photochemical applications.

**Figure 5:**
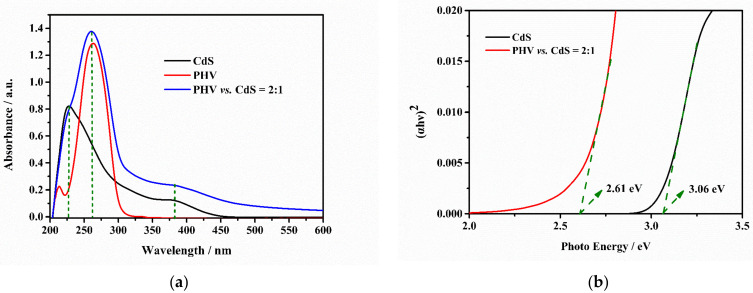
(**a**) UV-vis spectra of CdS, PHV, and PHV/CdS and (**b**) Tauc plots of CdS and PHV/CdS.

UV-vis spectroscopy was used to analyze the absorbances of the CdS and the PHV/CdS hybrid film. As is shown in Figure 5a, the absorbance spectra of the CdS, PHV, and PHV/CdS were measured from 200 nm to 600 nm. It was found that the PHV had a strong absorbance peak at approximately 265 nm, which was assumed to be the result of the π–π* transition of the pyridine rings from the viologens. In contrast, the CdS had a strong absorbance peak at approximately 225 nm, which was believed to be due to intrinsic defects in the CdS nanoparticles, e.g., their sulfur vacancy (VS) and sulfur gap (IS). Additionally, a weak absorbance peak was observed at approximately 380 nm, which can be explained by the quantum restriction effect of the CdS nanocrystals, proving the successful preparation of the CdS nanoparticles. When the hybrid film was formed from PHV and CdS, its absorption peaks included the characteristic peaks of both materials. The band gap values of the CdS and PHV/CdS were calculated using Tauc plots [47,48], as shown in Figure 5b. The band gap of the CdS nanoparticles was found to be 3.06 eV, while the band gap of the hybrid film was about 2.61 eV. It is worth noting that the band gap extension of the CdS may be due to the different preparation method. However, due to the assistance of the PHV, the band gap of the PHV/CdS hybrid film was smaller than that of the CdS, indicating that the electrons generated from the CdS can be transferred more easily, thus enhancing the charge separation efficiency.

Fluorescence spectroscopy was used to study the photoluminescence performance of the CdS, PHV, and PHV/CdS hybrid film. As is shown in Figure S4, the excitation peaks of the CdS and PHV were around 610 nm and 520 nm, respectively. However, the excitation peak of the PHV/CdS hybrid film was at about 475 nm, and this may have been caused by the interaction between the CdS and the PHV during the measurement affecting the result. Additionally, during the measurement, the excitation wavelength was 380 nm, and this might have caused the charge separation of the hybrid film, further affecting the accuracy of its band gap measurement. Therefore, these factors need to be taken into consideration when analyzing the photoluminescence performance of the PHV/CdS hybrid film.

**Figure 6:**
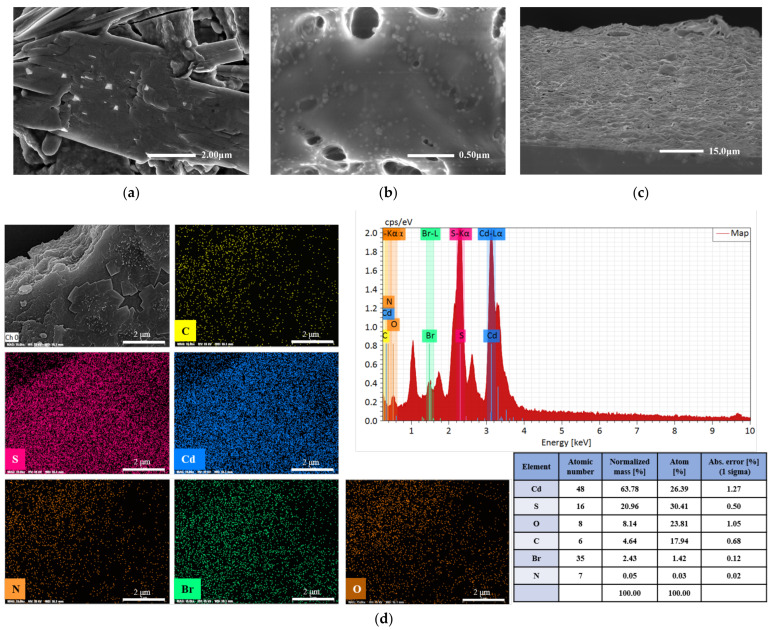
Electron microscopy analysis of the PHV/CdS hybrid films. (**a**,**b**) SEM images of PHV/CdS (**c**) SEM image of the thickness of the PHV/CdS and (**d**) EDX measurements and elemental mapping analysis of PHV/CdS.

Finally, SEM was utilized to analyze the morphology of the hybrid films, and Figure 6a shows the presence of CdS nanoparticles successfully immobilized on the surface of the PHV films. For the same reason we analyzed the distribution of CdS, the hybrid films were scratched and measured so that the inner side of the hybrid film could be shown. In Figure 6b, it can be seen that the CdS nanoparticles were immobilized in the film and that the distribution of CdS nanoparticles was fairly homogeneous, with no observed aggregation. Furthermore, the thickness of the PHV/CdS hybrid film was also measured using SEM, and the image is shown in Figure 6c. The PHV/CdS hybrid film was prepared, and the SEM image was taken from the side of the film. It can be observed that the film was well-defined and that the thickness was around 40–45 μm, proving that a PHV/CdS hybrid film with an even structure can be prepared using this simple method. Based on these results, we can conclude that the CdS nanoparticles were fairly well distributed in or on the surface of the hybrid film, enhancing the photoelectron transfer from the CdS to the PHV during photocatalysis applications.

Energy dispersive X-ray (EDX) measurements were used to analyze the microstructure and distribution of the CdS nanoparticles within the PHV/CdS hybrid film. Elemental mapping analysis was performed to determine the main elements present in the hybrid film, and the results are displayed in Figure 6d. The figure shows that the hybrid film contained several elements, including C, S, Cd, N, Br, and O, indicating that the PHV and the CdS were evenly distributed without any significant aggregation within the hybrid film. The information concerning each element is summarized in Figure 6d. This even distribution of CdS nanoparticles within the PHV/CdS hybrid film is essential for achieving optimal photoelectrochemical performance. When CdS nanoparticles are evenly distributed, they can efficiently generate photon electrons that can be transferred to the PHV for further reactions. This transfer of electron-hole pairs from the CdS to the PHV is critical for the overall performance of the hybrid material. If the CdS nanoparticles were only present on the surface of the PHV/CdS hybrid film, the photon electron transfer would be limited, and the overall photoelectrochemical performance would be reduced. Therefore, the EDX measurements and elemental mapping analysis provide important information about the distribution and microstructure of the CdS nanoparticles within the PHV/CdS hybrid film. These results confirm that the CdS nanoparticles were evenly distributed throughout the hybrid film, and this even distribution is crucial for achieving optimal photoelectrochemical performance.

## 4. Conclusions

In conclusion, a new PHV/CdS hybrid film with redox-enhanced photoelectrochemical properties was prepared using a simple and practical method. In this work, CdS nanoparticles and PHV were successfully synthesized and analyzed using XRD and NMR, respectively. After the electrochemical and photoelectrochemical analyses, the optimal ratio of the hybrid film with the best photoelectrochemical performance was determined to be 2:1. We demonstrated that the unique redox properties of PHV contributed to the enhanced photoelectrochemical performance of the hybrid film, confirming this using various characterization techniques, including EIS and UV-vis spectroscopy. The results of the SEM, TEM, and EDX analyses showed that the PHV/CdS hybrid film possesses a more homogeneous distribution of CdS nanoparticles, both on the surface and inside the film, resulting in improved photoelectron transfer efficiency from the CdS to the PHV during photocatalysis applications. Overall, this work offers a new hybrid film that has great potential for use in various photoelectrochemical applications in the future.

## Data Availability

Due to privacy concerns, we cannot share our data publicly. However, we are willing to provide the necessary data upon request for the purposes of scientific validation and reproducibility.

## Supplementary Materials

The following supporting information can be downloaded at: https://www.mdpi.com/article/10.3390/nano13091515/s1, Figure S1: XRD pattern of CdS; Figure S2: NMR of PHV; Figure S3: The cyclic voltammetry measurement of PHV/CdS hybrid film (2:1) before and after the photoelectrochemical analysis; Figure S4: Fluorescence of CdS, PHV and PHV/CdS hybrid film.
