# Supplementary material for: Redox-Enhanced Photoelectrochemical Activity in PHV/CdS Hybrid Film
Source: Nanomaterials (Basel). 2023 Apr 28;13(9):1515. doi: 10.3390/nano13091515 (PMC10180271; doi:10.3390/nano13091515)
Supplement: Supplementary file 1 [file nanomaterials-13-01515-s001.zip › nanomaterials-2357367-supplementary.pdf]

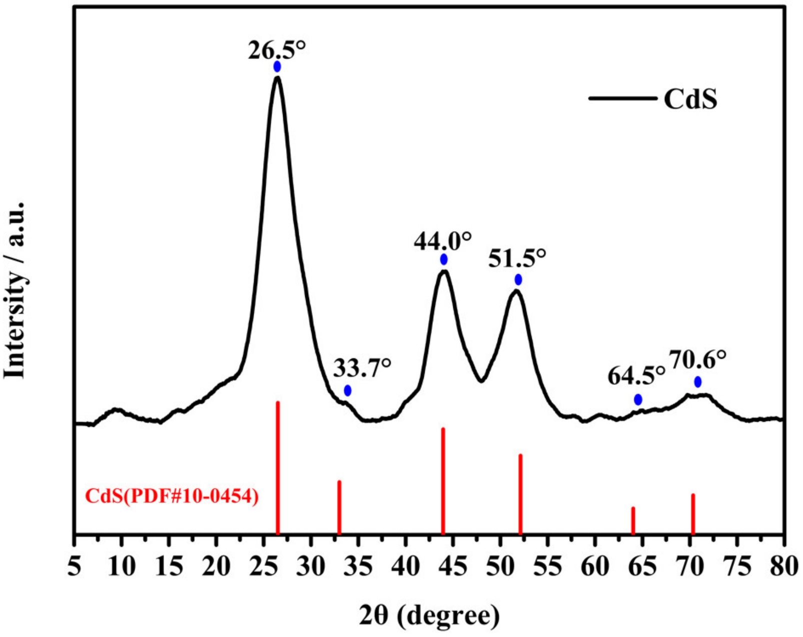

Figure S1. XRD pattern of CdS pnanoarticles.

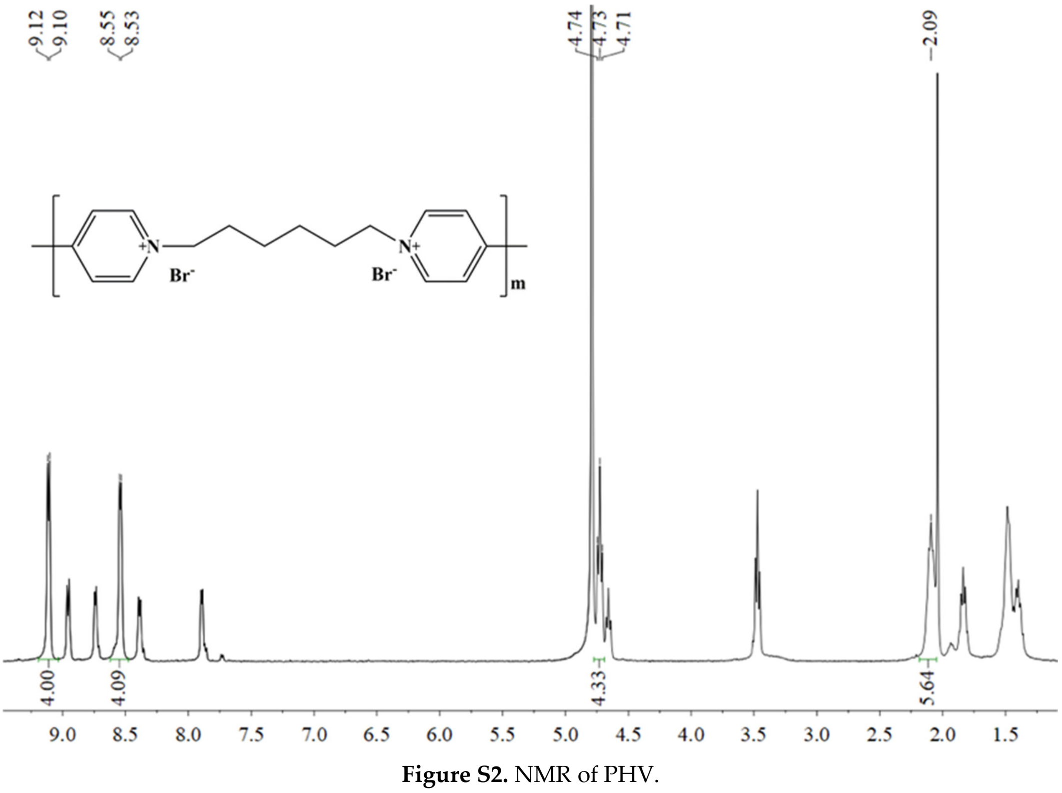

Figure S2. NMR of PHV.

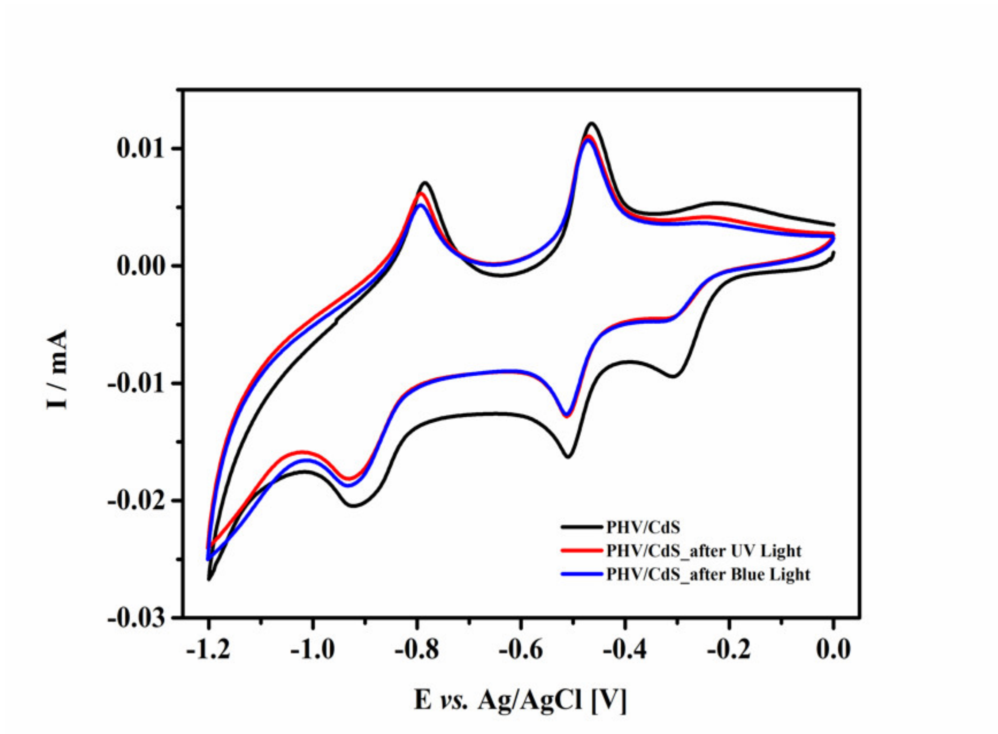

Figure S3. The cyclic voltammetry measurement of PHV/CdS hybrid film (2:1) before and after the photoelectrochemical analysis.

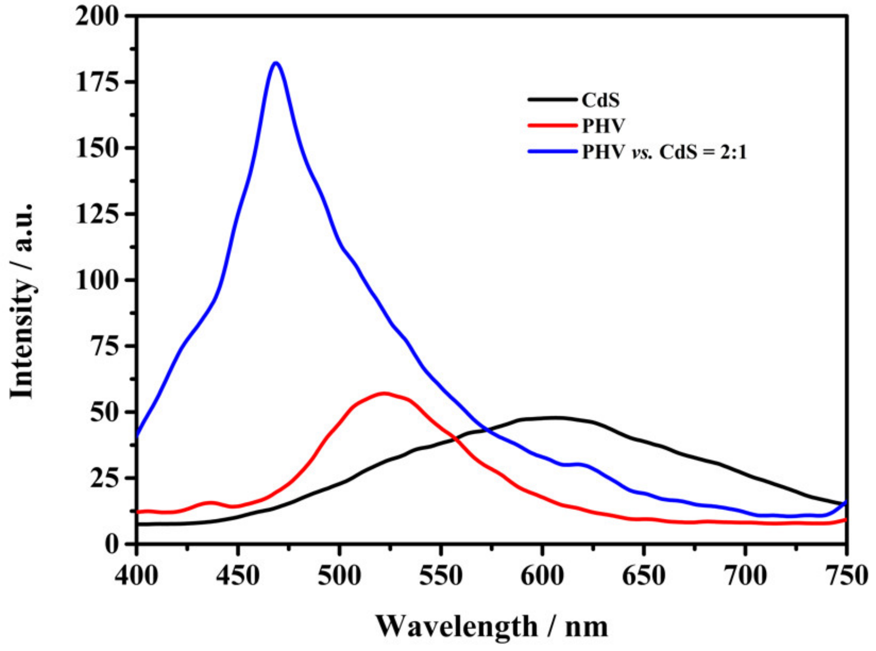

Figure S4. Fluorescence of CdS, PHV and PHV/CdS hybrid film.
